# Contact allergy to a titanium port catheter: Diagnostic challenges and clinical resolution 

**DOI:** 10.5414/ALX02637E

**Published:** 2026-07-01

**Authors:** Silas Fugger, Alexander Kauffmann, Heinrich Dickel

**Affiliations:** 1 Department of Dermatology, Venereology and Allergology, St. Josef Hospital, University Medical Center, Bochum, and; 2 Chair for Materials Science and Engineering, Institute for Materials, Faculty of Mechanical Engineering, Ruhr University Bochum, Bochum, Germany

**Keywords:** titanium allergy, contact sensitization, port catheter, medical device allergy, delayed-type hypersensitivity, allergy diagnostics, strip patch test, late patch test readings

## Abstract

Background: Titanium and its alloys are widely used in modern medical devices because of their favorable biocompatibility and mechanical properties. Allergic reactions to titanium-based implants are considered rare, and their diagnosis is hampered by the poor dermal penetration of metallic allergens. Case report: A 29-year-old female patient with seronegative myasthenia gravis requiring parenteral nutrition presented with persistent inflammatory dermatitis overlying a titanium port catheter (X-Port BARD Titan low-profile) 4 weeks after implantation in July 2023. Despite initial diagnostic uncertainty and sequential management modifications, her condition continued to deteriorate. Similar eruptions developed after re-implantation of the same titanium port model on the contralateral thorax. Rigorous patch testing incorporating standardized tape-stripping and extended reading intervals revealed positive sensitization to the titanium port body, while all other port components and an alternative plastic port system (BARD SlimPort M.R.I. Ultra Low-Profile) tested negative. Energy-dispersive X-ray spectroscopy confirmed the titanium body composition as a Ti-Al6-V4 alloy. Substitution with the plastic port catheter in February 2025 resulted in complete symptom resolution, with sustained clinical remission through July 2025. Conclusion: Although very uncommon, contact sensitization to titanium medical devices can occur and may present significant diagnostic and therapeutic challenges. Rigorous patch testing methodology, incorporating standardized tape-stripping and extended 168-hour readings, appears essential for detecting delayed-type hypersensitivity to metallic implants. Clinical resolution following biocompatible device substitution supports an allergic etiology in this case.

## Introduction 

Titanium and titanium alloys occupy a prominent position in modern medical technology due to their exceptional material properties [1], including excellent biocompatibility, high mechanical strength, superior corrosion resistance, radiopacity, and magnetic resonance imaging (MRI) compatibility. These characteristics render titanium alloys, particularly Ti-Al6-V4, the gold standard for diverse medical applications, including orthopedic implants, dental prostheses, cardiac devices, and central venous access systems [2]. The clinical tolerability of titanium materials is documented as excellent, with adverse reactions remaining exceptionally rare [3]. Allergic or intolerance reactions to medical titanium implants constitute a diagnostic rarity [4]. 

To our knowledge, we present the first documented case in the international medical literature of contact allergy to a titanium port catheter, confirmed by positive patch testing and clinical resolution following device substitution. 

## Case report 

### Medical history and clinical findings 

In December 2024, a 29-year-old female patient presented to our allergy department for evaluation of a suspected contact allergic reaction to her implanted port catheter or adhesive patches. The patient carried a diagnosis of seronegative myasthenia gravis accompanied by chronic dysphagia, necessitating partial parenteral nutrition for nutritional maintenance. A titanium port catheter (X-Port BARD Titan low-profile, BD, Heidelberg, Germany) was surgically implanted in the left anterior thorax in July 2023. 

**Figure 1:**
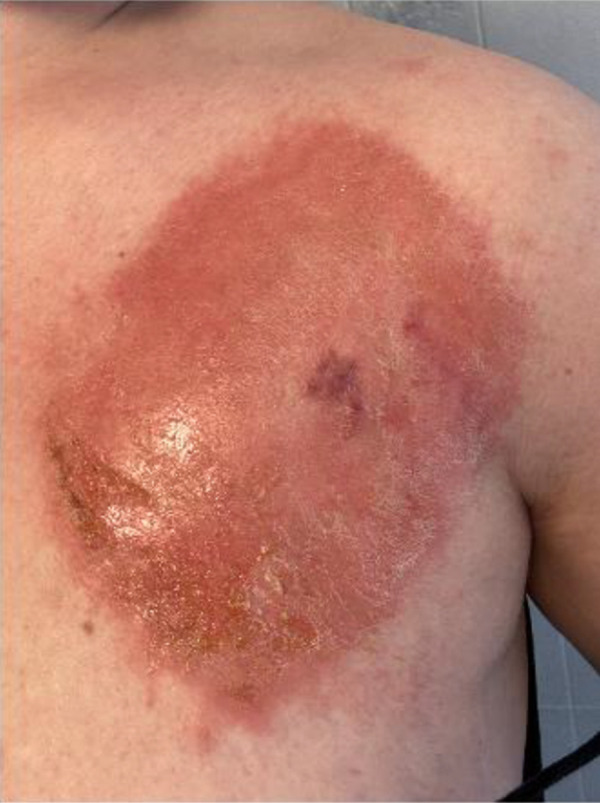
Inflammatory dermatitis overlying the implanted titanium port catheter in the left anterior thorax. Presentation at 4 weeks post-implantation of X-Port BARD Titan low-profile catheter (BD, Heidelberg, Germany). Characteristic findings include sharply demarcated erythematous plaques with marginal scaling, vesiculation, and centrifugal spread, accompanied by substantial pruritus.

**Figure 2:**
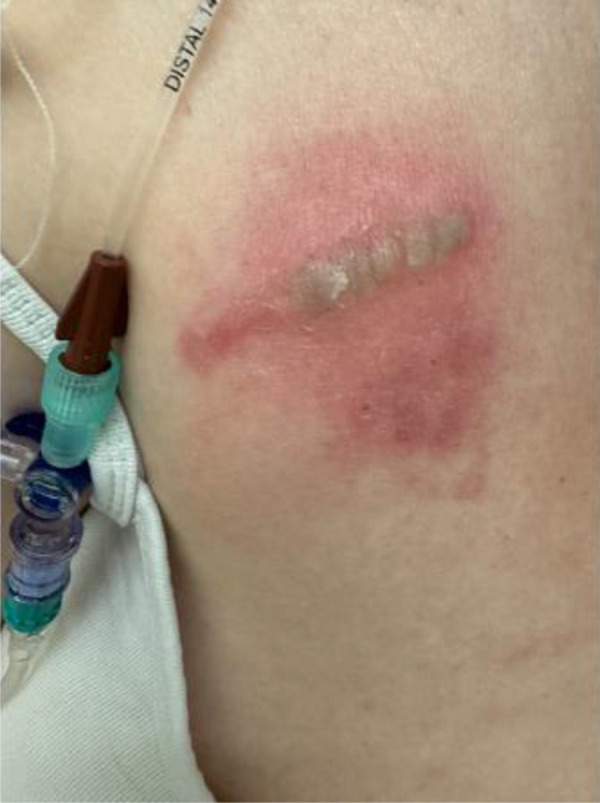
The skin overlying the re-implanted titanium port catheter on the right anterior thorax, demonstrating identical inflammatory eruption in the postoperative period. Note the hypertrophic implantation scar from the initial surgical procedure and the recurrence of sharply demarcated erythema with scaling and vesiculation, confirming the association between the port implantation and the dermatitis.

Approximately 4 weeks post-implantation, the patient developed visible inflammatory skin changes overlying the port chamber, characterized by sharply demarcated erythematous plaques with associated papules, vesicles, and scaling, accompanied by significant pruritus (Figure 1). Initial diagnostic consideration suspected a contact dermatitis from adhesive patches utilized for port fixation. Subsequently, adhesive patches were changed repeatedly, and periods of patch cessation were trialed, yet the inflammatory reaction continued to intensify. Following a port infection complicated by sepsis several months post-implantation, surgical explantation was performed. The identical titanium port model was then re-implanted on the contralateral (right) side, whereupon an identical inflammatory skin eruption became clinically apparent in the postoperative period (Figure 2). 

At the time of initial allergological consultation, the patient had experienced absence of permanent vascular access for 3 months, resulting in significant nutritional compromise and clinical urgency regarding diagnostic clarification and therapeutic resolution. 

### Allergological diagnostics 

**Patch testing and initial findings**

**Figure 3:**
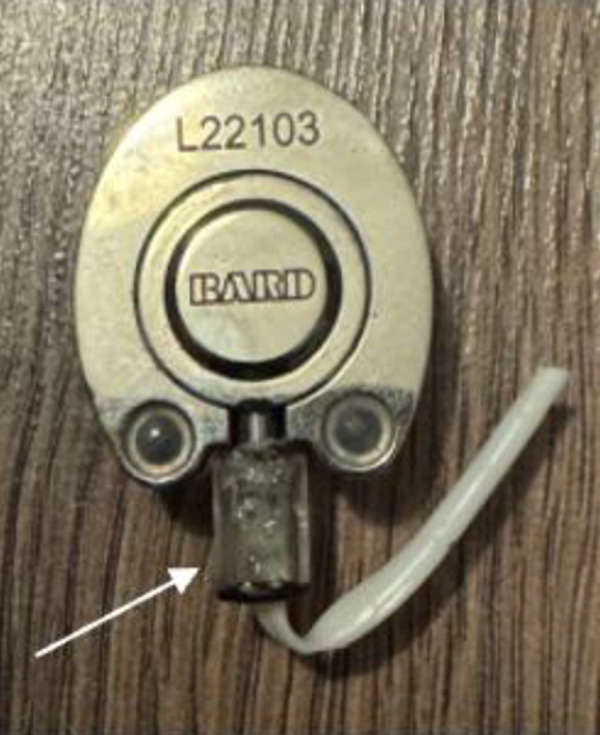
Explanted titanium port system (X-Port BARD Titan low-profile, BD, Heidelberg, Germany), posterior view, demonstrating the port body, connector (indicated by arrow), and attached catheter. This component was subjected to patch testing following standardized tape-stripping, revealing positive sensitization to the port body.

Comprehensive patch testing was undertaken utilizing standardized test series from the German Contact Allergy Group (DKG), including the standard series, external ingredients, preservatives, disinfectants, rubber compounds, dental metals (with preliminary standardized tape-stripping [5]), and synthetic resins/adhesives. Additionally, patch testing with the patient’s own explanted port system, including the port body, connector, and catheter (Figure 3), was performed, with separate testing of the puncture membrane and port needle following standardized tape-stripping. Readings were conducted at 24 hours (where standardized tape-stripping had been performed), and at 48, 72, and 168 hours. Initial testing revealed positive reactions to tert-butylhydroquinone and, at the 168-hour reading, to the port system itself. 

**Figure 4:**
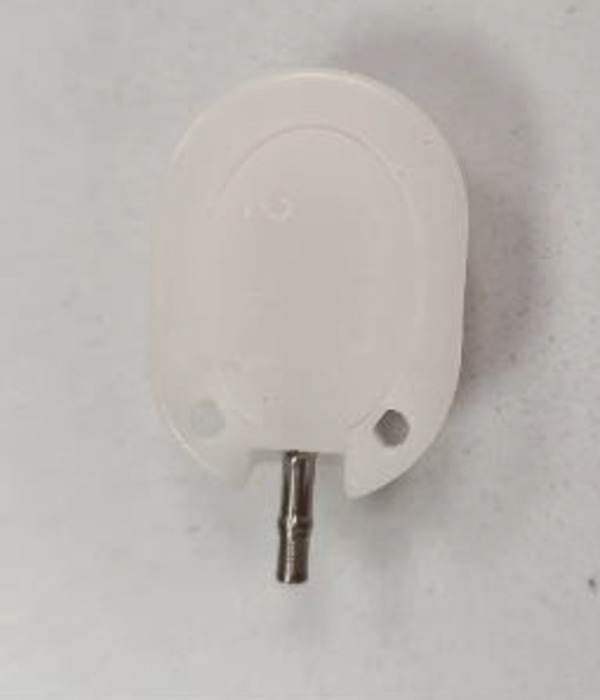
Alternative plastic port system (BARD SlimPort M.R.I. Ultra Low-Profile, BD, Heidelberg, Germany): underside of the port housing. All components of this plastic port system tested negative in patch testing and were utilized for successful substitution therapy.

**Figure 5:**
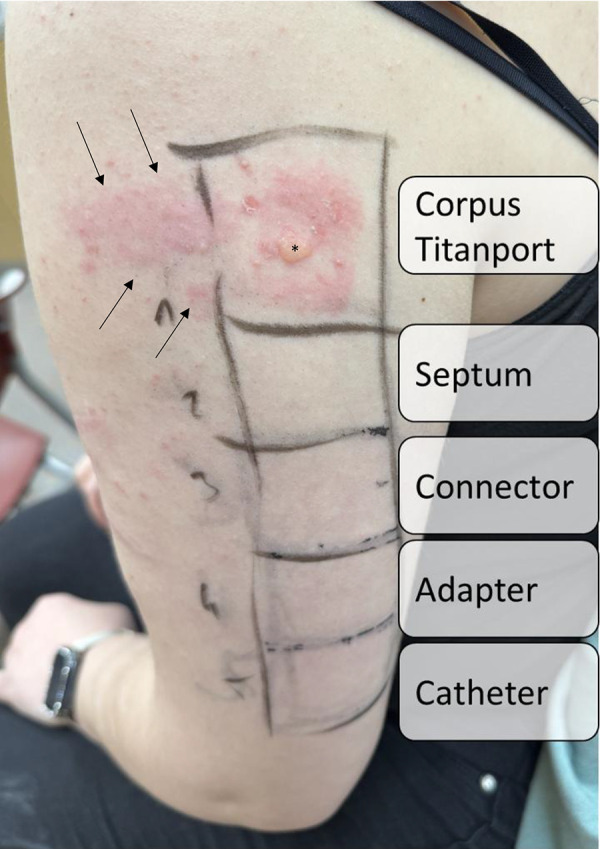
Patch test on the left upper arm, reading 168 hours after application. Examination reveals a strongly positive reaction at the test site of the titanium port body, characterized by marked erythema, induration, papulation, and centrifugal spread of the reaction (indicated by arrows). Confluent vesicles are evident at the site of immediate port body contact (indicated by asterisk). All other test areas, including individual port components and plastic port materials, demonstrated negative reactions. The polycarbonate adapter tested is not a direct component of the port system proper.

Component-specific and comparative testing: Given the suspected port-related contact sensitization, individual components of both the titanium port catheter and an alternative plastic port catheter (BARD SlimPort M.R.I. Ultra Low-Profile, BD) (Figure 4) underwent sequential testing following standardized tape-stripping (Table 1). At 168 hours, a distinctly positive skin reaction characterized by marked erythema, infiltration, papules, and centrifugal spread with confluent vesiculation at the immediate contact point was observed exclusively at the test site of the titanium port body (Figure 5). All other individual port components and all components of the plastic port system demonstrated negative reactions. 

**Materials analysis**

To identify potential allergenic components within the port housing, energy-dispersive X-ray spectroscopy (EDS) was performed using a scanning electron microscope. This analysis definitively confirmed the port body composition as a Ti-Al6-V4 alloy (titanium 90%, aluminium 6%, vanadium 4%) with only minor trace impurities of sodium, silicon, and calcium. Notably, tert-butylhydroquinone, which demonstrated patch test positivity in initial screening, was not detected on the port body surface by EDS analysis and therefore could not represent the causative allergen. 

**Differential diagnostic considerations**

Aluminium sensitization was considered unlikely, as the aluminium-containing Finn Chamber test chambers did not elicit reactions at other test sites [6]. Further specific patch testing with titanium 1% pet or vanadium pentoxide 10% pet. was not performed because the patient declined such testing due to mobility limitations and it was not considered clinically relevant for her management. Noteworthy, neither of the two test preparations had been recommended by the DKG for routine testing in one of its patch test series. 

**Clinical course and resolution**

Following confirmation of titanium contact sensitization, a plastic port catheter (BARD SlimPort M.R.I. Ultra Low-Profile) was surgically implanted in February 2025 to provide continued vascular access while eliminating the titanium allergen source. The patient achieved complete clinical remission of the inflammatory dermatitis, remaining entirely symptom-free throughout the subsequent period of port use. The port required removal in July 2025 due to bacteremia-related complications. Subsequently, a metal-free Broviac catheter was implanted as a permanent vascular access option, selected due to its substantially lower infection risk profile during daily utilization compared to port catheter systems. 

## Discussion 

This case describes the first documented detection of contact allergic reaction to a titanium port catheter housing, representing, to our knowledge, the first case report in the international medical literature of proven contact sensitization to a titanium port with definitive patch test confirmation and clinical resolution following device substitution. This presentation carries particular significance within clinical allergy, given the ubiquitous and expanding utilization of titanium-based medical devices across diverse healthcare applications. The clinical dilemma presented by this case, in which an essential therapeutic intervention provokes a contact allergic reaction, underscores the importance of rigorous allergological diagnostics in enabling continuation of necessary medical therapy through biocompatible alternative devices. 

The Ti-Al6-V4 alloy employed in this patient, alongside commercially pure titanium, currently represents the most frequently utilized material for medical implants across diverse clinical disciplines. Documented reports and case series of allergic reactions to titanium implants have been published across dentistry, orthopedics, trauma surgery, neurosurgery, and cardiovascular surgery, with reported symptom presentations ranging from prosthesis intolerance with implant loosening and localized inflammatory reactions to systemic manifestations including fever, malaise, DRESS syndrome (drug reaction with eosinophilia and systemic symptoms), and granulomatous dermatitis [7]. Nevertheless, such allergic phenomena remain exceptionally uncommon, reflecting the generally excellent biocompatibility profile of titanium materials in clinical practice. 

The diagnostic challenges inherent in detecting contact sensitization to metallic medical devices merit substantial emphasis. Traditional in vitro diagnostic modalities, including lymphocyte transformation tests (LTT) and refined variants thereof, remain inadequately validated for metal allergy diagnostics and demonstrate variable sensitivity and specificity. Patch testing remains the diagnostic gold standard for contact allergy; however, detection sensitivity is frequently compromised by intrinsic technical limitations, specifically the poor dermal penetration of metallic allergens and the low bioavailability of metallic ions at the skin surface [8]. 

The exact pathophysiological mechanism of titanium allergy remains incompletely understood, but proposed mechanisms include reactive titanium oxide intermediates, impurities within the alloy, and trace metallic components released through corrosion or wear. In this case, aluminium sensitization appears unlikely given the absence of reactions at other aluminium-containing test sites, and vanadium allergy, although documented, is rare, typically associated with orthopedic prostheses rather than port systems [9] and less probable here in view of the alloy’s low vanadium content (4%). Taken together with the 4-week latency before onset, recurrence after re-implantation, and complete remission after device removal, the findings are most consistent with a titanium-driven type IV contact hypersensitivity reaction within the Ti-Al6-V4 alloy. 

Successful diagnostic confirmation in this case was attributable to two critical methodological modifications to conventional patch testing:

- Standardized tape-stripping prior to patch testing (the so-called strip patch test [5]): this technique enhances percutaneous allergen penetration by removing stratum corneum barrier layers, thereby augmenting allergen bioavailability and substantially increasing sensitivity for detecting metallic contact sensitization.
- Extended reading intervals: reading patch test results at 168 hours (after 7 days), as recommended by current guidelines for metallic contact allergens [10], permitted detection of delayed-type hypersensitivity reactions that would not manifest at standard 48- or 72-hour reading intervals.

However, the ultimate diagnostic confirmation was the clinical improvement consequent upon titanium port removal and the complete resolution of symptoms following substitution with a non-allergenic plastic port, representing the most definitive evidence for titanium device-mediated allergic etiology. 

## Conclusion 

Although exceptionally uncommon, delayed-type contact sensitization to titanium medical devices must be considered in patients presenting with unexplained inflammatory skin reactions adjacent to titanium implants. Rigorous patch testing incorporating standardized tape-stripping and extended reading intervals can facilitate diagnosis, and clinical resolution can be achieved through substitution with alternative biocompatible materials (such as plastics and polymers). 

## Acknowledgment 

We gratefully acknowledge our patient for her willingness to participate in this case publication and to undergo comprehensive allergological investigations despite her significant mobility limitations. 

## Authors’ contributions 

SF and HD conceived and designed the clinical allergological evaluation. SF collected clinical data and obtained clinical photographs. AK performed and interpreted energy-dispersive X-ray spectroscopy analysis. HD supervised allergological diagnostics and patch testing protocols. SF wrote the manuscript. All authors reviewed and approved the final manuscript. 

## Funding 

All authors declare that no funding was received for the present work. 

## Conflict of interest 

All authors declare that they have no conflict of interest relevant to this work. 

**Table 1.** Component composition and materials specification of titanium and plastic port systems according to manufacturer designation.

**Component**	**Titanium port system**	**Plastic port system**
Port body	Titanium, vanadium, aluminium (Ti-Al6-V4 alloy)	Delrin thermoplastic
Septum (puncture membrane)	Silicone	Silicone
Catheter	Polyurethane with 20% barium sulphate	Polyurethane with 20% barium sulphate
Connector	Polycarbonate	Polycarbonate
Connector marking	99.9% pure tungsten metal powder	99.9% pure tungsten metal powder
Catheter markings	Black ink	Black ink
